# Retinoic Acid and Rapamycin Differentially Affect and Synergistically Promote the *Ex Vivo* Expansion of Natural Human T Regulatory Cells

**DOI:** 10.1371/journal.pone.0015868

**Published:** 2011-01-06

**Authors:** Tatiana N. Golovina, Tatiana Mikheeva, Todd M. Brusko, Bruce R. Blazar, Jeffrey A. Bluestone, James L. Riley

**Affiliations:** 1 Department of Pathology and Laboratory Medicine and Abramson Family Cancer Research Institute, University of Pennsylvania, Philadelphia, Pennsylvania, United States of America; 2 The Diabetes Center at the University of California, San Francisco, California, United States of America; 3 University of Minnesota Cancer Center and Division of Bone Marrow Transplant, Department of Pediatrics, University of Minnesota, Minneapolis, Minnesota, United States of America; New York University, United States of America

## Abstract

Natural T regulatory cells (Tregs) are challenging to expand *ex vivo*, and this has severely hindered *in vivo* evaluation of their therapeutic potential. All *trans* retinoic acid (ATRA) plays an important role in mediating immune homeostasis *in vivo*, and we investigated whether ATRA could be used to promote the *ex vivo* expansion of Tregs purified from adult human peripheral blood. We found that ATRA helped maintain FOXP3 expression during the expansion process, but this effect was transient and serum-dependent. Furthermore, natural Tregs treated with rapamycin, but not with ATRA, suppressed cytokine production in co-cultured effector T cells. This suppressive activity correlated with the ability of expanded Tregs to induce FOXP3 expression in non-Treg cell populations. Examination of CD45RA+ and CD45RA− Treg subsets revealed that ATRA failed to maintain suppressive activity in either population, but interestingly, Tregs expanded in the presence of both rapamycin and ATRA displayed more suppressive activity and had a more favorable epigenetic status of the *FOXP3* gene than Tregs expanded in the presence of rapamycin only. We conclude that while the use of ATRA as a single agent to expand Tregs for human therapy is not warranted, its use in combination with rapamycin may have benefit.

## Introduction

FOXP3-expressing, natural Tregs cells play a crucial role in maintaining immune homeostasis by attenuating aberrant immune responses. Humans and mice lacking a functional FOXP3 gene suffer from massive, multi-organ, usually fatal autoimmunity [39]. Given the central role Tregs play in immune control, it has long been postulated that adoptive transfer of functional Tregs could be a potent weapon in the fight against autoimmune disease, graft v. host disease (GVHD), and transplant rejection [1]. The availability of inbred mouse strains and an easily identifiable population of CD4^+^CD25^+^ Tregs have enabled numerous proof-of-principle experiments demonstrating that, in mice, adoptively transferred natural Tregs can treat type 1 diabetes [44], prevent GVHD [45], [21], and increase the success of organ transplantation [12].

The transition from murine studies to human Phase I Treg clinical trials has been difficult, largely due to difficulties in isolating and expanding Tregs in an Good Manufacturing Practice (GMP) manner (reviewed in Riley et al [38]). An additional, and perhaps even more important issue, centers on the plasticity of the Treg phenotype. In the right cytokine milieu, a small percentage of natural Tregs stop expressing FOXP3 and start expressing inflammatory agents such as IL-17 and IFN-γ [27], [51]. In mice, adoptive transfer of these ex-Tregs led to the rapid onset of type 1 diabetes [53]. This raises the significant safety concern that expanded Tregs targeted to a specific tissue may do more harm than good if substantial numbers of them differentiate into inflammatory/effector cells. Given the seriousness of these issues regarding the stability and function of *ex vivo* expanded Tregs, many investigators have sought culture conditions that promote the stable expansion of fully functional natural Tregs. These investigations have focused on identifying costimulatory pathways and soluble reagents that best enable expansion of functional human Tregs. For example, we previously showed that CD28 costimulation was absolutely necessary for large-scale expansion of functional human Tregs [18]. With respect to soluble agents that favor human Treg expansion, rapamycin has emerged as the agent of choice to date . Rapamycin-mediated mTOR inhibition blocks critical T cell effector functions such as migration and cytokine production, and limits T cell expansion. However, Tregs appear to function effectively in the presence of rapamycin [6]. Importantly, FOXP3 expression induces the serine/threonine kinase pim-2 [5], which permits Tregs to evade many rapamycin-imposed signaling blocks [17]. Thus, Tregs are endowed with a natural resistance to rapamycin, allowing them to preferentially expand in its presence. Nonetheless, rapamycin has drawbacks. While it provides a selective advantage for Treg growth, it still impairs overall Treg expansion [7]. This may necessitate extended *ex vivo* culture and/or multiple rounds of T cell activation in order to generate therapeutic levels of Tregs. Additionally, rapamycin has been shown to promote memory cell formation [3] which may translate into long term side effects of persistent memory Tregs in some adoptive Treg cell therapy applications. These drawbacks have provided the rationale to search for agents that will equal or better rapamycin in terms of generating pure expanded Treg populations, while eliminating the rapamycin-imposed decrease in overall Treg expansion.

Retinoic acid, a derivative of vitamin A, plays an important role in T cell function and trafficking and has been postulated as an alternative to rapamycin to promote the expansion of Tregs. ATRA produced by DCs facilitates de novo generation of FoxP3^+^ T regulatory cells from CD25^−^ T cell populations in mice [13], [43]. Two non-mutually exclusive mechanisms have been proposed to account for the ATRA-promoted induction of suppressive T cells. One set of data indicates that ATRA augments TGF-β mediated signaling [8], [50], while other investigators report that ATRA suppresses the ability of memory T cells to block the induction of FoxP3 expressing regulatory T cells [19]. Many studies have tried to mimic the *in vivo* conversation of Teffs to Tregs by ATRA *in vitro* as means to rapidly generate suppressive T cells [33], [40], [15]. These induced (i)Tregs are attractive immunotherapeutic agents because of their ease of generation and their potent function in murine models of autoimmunity, but questions remain regarding their stability [10]. Two studies have reported that human CD4+CD25− T cells derived from adult peripheral and cord blood were converted to suppressive cells in the presence of TGF-β and ATRA [24], [48]. One of these groups also performed a series of experiments examining how ATRA affected the expansion and function of natural Tregs and concluded that ATRA augmented their suppressive activity and should therefore be considered for use in Treg-based therapy.

Our studies, in which we amplified natural Tregs *ex vivo* in the presence of ATRA, came to different conclusions. We found that while ATRA did promote FOXP3 expression and suppressive activity, the effects were transient. Our studies evaluating the stability of ATRA-cultured human Tregs indicate that ATRA has no long-term beneficial effect as a single agent and may actually be detrimental to generating Tregs with durable activity. Furthermore, our studies further highlight that criteria other than *in vitro* suppression and FOXP3 expression are required to demonstrate Treg function and stability. To this end, we provide further data supporting the analysis of the methylation status of the Treg-specific demethylated region (TSDR) within the non coding region of *FOXP3* following extended *in vitro* culture as an indicator of Treg function and stability. Lastly, the addition of ATRA to rapamycin-treated cultures improved FOXP3 expression, resulted in less methylation at the *FOXP3* TSDR region, and improved Treg suppressive activity. These observations suggest that ATRA may have utility in *ex vivo* expansion of human Tregs, not as a single agent, but in combination with rapamycin.

## Results

### ATRA does not impair human T cell proliferation and augments FOXP3 expression during ex vivo expansion

**Figure 1:**
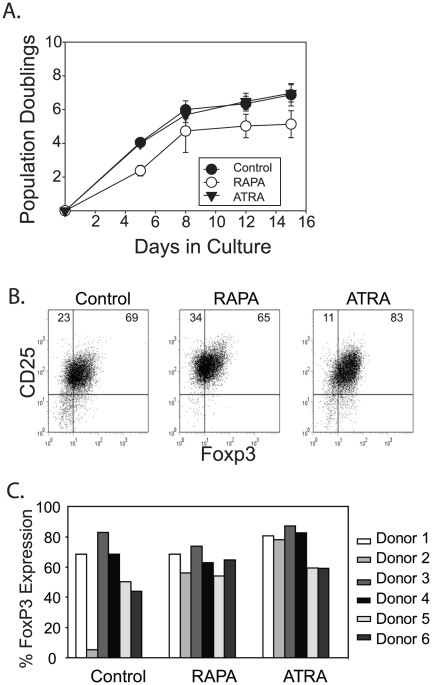
ATRA does not impair human T cell proliferation and augments FOXP3 expression during *ex vivo* expansion. **A**. 200,000 sorted primary human Tregs were stimulated with anti-CD3 Ab loaded K.64.86 cells and cultured in the presence of 10% human serum alone for 15 days (control) or in serum-containing medium with the addition of 100 ng/ml of rapamycin (RAPA) or 10 µM ATRA. Cell counts were performed on Days 5, 8, 12 and 15 and data reflect the average of 3 independent experiments. The error bars reflect standard deviation. **B**. After 15 days of culture, each cell population from a single donor described in Panel A was analyzed for CD25 and FOXP3 expression by flow cytometry. **C**. Composite FOXP3 expression data from 6 independent experiments performed using 6 unique donors. The donor shown in Panel B is donor #4.

The greatest drawback to using rapamycin to culture and expand Tregs is that the overall Treg yield is disappointing. Before investigating ATRA-specific effects on Tregs, we determined whether ATRA affected overall Treg expansion. Based on previous work [2], [30] and our own preliminary experiments, we found that ATRA exerted effects over a wide concentration range (10nM–1µM). For our initial studies we added 1 µM ATRA or our previously defined optimal concentration of rapamycin (100nM) to purified CD4^+^, CD25^+^, CD127^dim^ Tregs, activated them using KT64.86 cells loaded with anti-CD3 Abs, and monitored cell expansion for 15 days. As previously described , we observed less T cell expansion in cultures containing rapamycin (**Fig 1A**). Decreased Treg yields were not observed when human Tregs were expanded in the presence of ATRA, supporting the notion that ATRA could overcome the major drawback of using rapamycin to promote *ex vivo* Treg expansion. It should be noted that Tregs expanded in the presence of both RAPA and ATRA grew similarly to cells cultured in the presence of RAPA alone, indicating that the anti-proliferative effects of RAPA were not overcome by ATRA (data not shown).

We next examined FOXP3 expression in the expanded T cell populations (**Fig 1B**). Overall, the addition of either ATRA or RAPA appeared to slightly augment FOXP3 expression in expanded Treg cells (**Fig 1C**), although this difference did not reach statistical significance (P = .123 ANOVA for all groups; P = .08 comparing control and ATRA treated Treg populations). Notably, purified Tregs from one donor (donor 2) failed to maintain FOXP3 expression through the two week expansion period. However, when these same cells were expanded in the presence of ATRA or RAPA, they maintained high levels of FOXP3, suggesting that both RAPA and ATRA could help maintain the Treg phenotype during *ex vivo* expansion (Fig 1C). These studies suggest that ATRA, like RAPA, enforces a Treg phenotype during *ex vivo* expansion.

### Tregs undergoing more than one round of ex vivo expansion in the presence of ATRA do not maintain their suppressive activity

**Figure 2:**
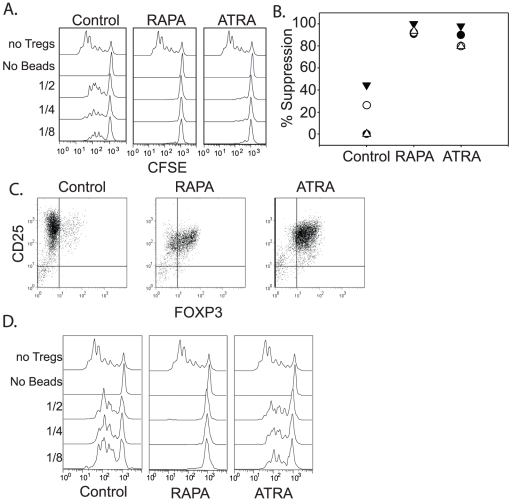
Tregs undergoing more than one round of ex vivo expansion in the presence of ATRA do not maintain their suppressive activity. **A**. Highly enriched Tregs expanded for 12–15 days under control conditions, or in the presence of ATRA or RAPA as described in Fig 1, were tested in a suppression assay. Autologous PBMCs were labeled with CFSE, stimulated using anti-CD3 Ab coated beads, and mixed with either no Tregs (top panel) or expanded Tregs at Treg∶PBMC ratios of 1∶2, 1∶4 or 1∶8. Histograms show the expansion of CD8+ T cells. PBMCs that did not receive anti-CD3 Ab coated beads were used a negative control. **B**. Suppressive activity of cultured Tregs from 4 different donors was calculated as described in the Method and Materials Section. Depicted are the % suppression values obtained from the 1∶4 Treg∶Eff cultures. **C**. and **D**. After a single round of expansion, Tregs were restimulated using anti-CD3 loaded K.64.86 cells and cultured under the same conditions as during the first stimulation. After an additional 12 days of culture, FOXP3 expression was analyzed by flow cytometry (**C**.), and a suppressive assay was performed and analyzed as described above (**D**.). FOXP3 data is representative of 3 donors, and the suppressive assay data is representative of 2 of 3 cultures. One donor maintained suppressive activity in the presence of ATRA treatment (data not shown).

Next, we tested the ability of the ATRA-expanded Tregs to function as suppressive cells. After 10–12 days of culture, the expanded Treg populations were mixed at varying ratios with CFSE-labeled autologous PBMCs and anti-CD3 Ab coated beads. After 4–5 days of co-culture, the ability of expanded Tregs to limit expansion of the CFSE labeled CD8 T cells was measured by flow cytometry (**Fig 2A**). Control Tregs expanded in the absence of RAPA and ATRA displayed highly variable suppressive activity, whereas those expanded in the presence of ATRA or RAPA uniformly maintained high suppressive activity (**Fig 2B**). Previously, we demonstrated that Tregs expanded in the presence of rapamycin could be restimulated using aAPCs coated with anti-CD3/28 Abs and cultured for an additional 10–12 days without losing their ability to function *in vitro* and *in vivo*. These additional restimulations may be necessary in scenarios in which there is only limited amount of starting Tregs (cord blood) or when multiple infusions of Tregs will be necessary to observe a therapeutic effect. We restimulated ATRA-expanded Tregs with anti-CD3 Ab-loaded K.64.86 cells to determine whether ATRA-expanded Tregs could also maintain their suppressive phenotype after an additional round of expansion in the presence of ATRA. Restimulated Tregs cultured in the absence of either RAPA or ATRA had few FOXP3+ cells remaining after 24–28 days of culture. This decrease could be attributed to outgrowth of contaminating effectors and/or the plasticity of the Treg phenotype in the absence of selective agents. In contrast, Tregs cultured with either RAPA or ATRA maintained high levels of FOXP3 expression following restimulation (**Fig 2C**). However, unlike what was observed with rapamycin-treated Tregs, 2 of 3 ATRA-treated Treg cultures completely lost their suppressive activity after a single round of restimulation and culture, while RAPA-treated Tregs robustly suppressed effector cell division in all cultures (**Fig 2D**, and data not shown). These data indicate that ATRA does not promote the expansion of cells with a durable Treg phenotype, and furthermore, these data emphasize the difficulties of using FOXP3 expression as the sole surrogate for Treg activity.

### Treg expansion performed in the absence of serum results in more durable Treg suppressive activity

**Figure 3:**
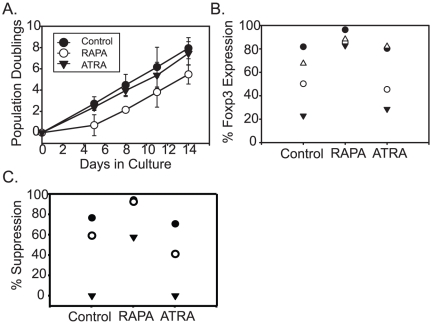
Treg expansion performed in the absence of serum results in more durable Treg suppressive activity. **A**. Enriched Tregs were expanded in the absence of human serum. Where indicated, 10 nM ATRA or 100 ng/ml RAPA were added to the medium and this concentration of drug was added to media used to feed the cultures. Cell counting was performed on the indicated days. The data reflects the average of 3 cultures using enriched Tregs from different healthy donors. Error bars represent the standard deviation. (**B**.) At the end of culture, FOXP3 expression was measured by flow cytometry, and (**C**.) an *in vitro* suppressive assay was performed. For parts B. and C., each symbol refers to data collected from a single donor.

We were interested in understanding how ATRA could induce a transient but not a sustained Treg phenotype. Several groups have reported that ATRA requires TGF-β to augment FOXP3 expression [32]. The role of TGF-β in promoting Treg differentiation differs significantly between human and murine Tregs. TGF-β is necessary and sufficient to convert naïve murine CD4 T cells into suppressive T cells, while TGF-β induces FOXP3 expression, but not suppressive activity, in naïve human CD4T cells [46]. Thus, we suspected that TGF-β present in the human serum used in Treg culture medium may have contributed to the transient suppressive Treg phenotype observed in Tregs expanded in ATRA. To test this, we expanded Tregs in the absence of human serum. We found it necessary to repeat ATRA and RAPA dose titrations to determine their optimal concentrations in serum-free medium formulations. ATRA concentrations above 10 ng/ml were highly toxic to human Tregs cultured in serum-free conditions (data not shown), whereas the optimal RAPA concentration remained at 100ng/ml. Thus, ATRA toxicity, but not RAPA toxicity, is altered by the presence of human serum. Next, Tregs isolated from three consecutive healthy donors were cultured for one stimulation cycle only under serum-free conditions in the presence of ATRA or RAPA. Remarkably, control Treg cultures expanded similarly under both serum-free and serum-containing conditions (compare **Fig 1A** with **Fig 3A**). Additionally, we observed that RAPA inhibited Treg expansion, but ATRA did not, in agreement with our results obtained using human serum-containing culture medium. FOXP3 levels were virtually identical between control and ATRA-treated cultures grown in the absence of serum, whereas RAPA-treated cultures had higher levels of FOXP3 expression than controls or ATRA-treated Tregs when grown in the absence of serum (**Fig 3B**, P = 0.01 One-Way ANOVA). Finally, ATRA-treated Tregs grown in the absence of serum exhibited poor suppressive activity compared to Tregs grown in RAPA (**Fig 3C**). Thus, these studies suggest that *ex vivo* expansion of Tregs should be performed in the absence of serum since under certain circumstances, serum promotes the expansion of T cells that have transient Treg phenotype and function.

### Tregs expanded in the presence of RAPA, but not ATRA, can induce infectious tolerance

**Figure 4:**
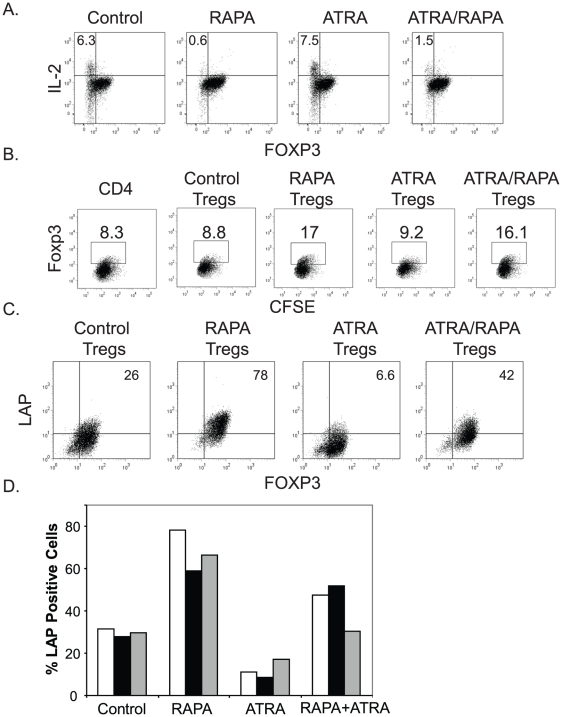
Tregs expanded with RAPA, but not ATRA, induce infectious tolerance. **A**. Enriched Tregs were expanded in the presence of the indicated compounds. At the end of the culture period (Day 15), the cells were stimulated with PMA+ ionomycin for 4 hrs and intracellular IL-2 and FOXP3 expression were analyzed by flow cytometry. **B**. CFSE labeled FOXP3-negative CD4 T cells were activated with anti-CD3 Ab coated beads and mixed 1∶1 with 2 day activated Tregs cultured with the indicated compounds. After 3 days of co-culture, cells were analyzed for FOXP3 expression by flow cytometry; the CFSE labeled cells are displayed. **C**. and **D**. Enriched Tregs were activated and expanded using the indicated culture conditions and expression of the Latency Associated Peptide (LAP) was determined after 4 days of culture by flow cytometry from a representative single donor (**C**.). The composite data from multiple (3) donors is portrayed in **D**.

Infectious tolerance refers to the ability of one cell population to induce a suppressive phenotype in another cell population [35]. As mentioned earlier, it is currently not possible to obtain a 100% pure population of FOXP3-expressing cells. Thus, in every expanded Treg culture, there is a population of non-FOXP3 expressing Teffs. We wished to determine whether Tregs cultured with ATRA and/or RAPA were capable of inducing infectious tolerance in non-FOXP3-expressing T cells. Our first set of experiments involved examination of expanded enriched Treg populations that contain a mixture of CD4+ FOXP3-negative Teffs and Tregs that had been expanded with either ATRA and/or RAPA. We treated the expanded co-cultures with PMA and ionomycin and examined intracellular IL-2 production to determine whether expanded Tregs could prevent effector cytokine production by the FOXP3 non-expressing Teff cells. As expected, the FOXP3-expressing cells were unable to produce IL-2 regardless of the culture conditions (**Fig 4A**). However, in the co-cultures containing untreated or ATRA-treated Tregs, a significant fraction of the FOXP3-negative CD4 T cells produced IL-2 after PMA+ ionomycin treatment. In co-cultures containing Tregs expanded with RAPA or RAPA plus ATRA, PMA+ ionomycin stimulation did not result in IL-2 production by the non-FOXP3 expressing cells, supporting the notion that RAPA-expanded Tregs can induce infectious tolerance.

These data suggested that RAPA− and ATRA-treated Tregs differed in their ability to induce infectious tolerance in Teffs, but other explanations, including the effects of the drugs themselves, could not be ruled out. To explore this differential induction of infectious tolerance in greater detail, we asked if ATRA− or RAPA-treated expanded Tregs could induce FOXP3 expression in Teffs. To do this, we mixed anti-CD3 Ab coated, bead activated, CFSE-labeled CD4 Teff cells with Tregs that had been activated with anti-CD3 Ab coated K.64.86 aAPCs and cultured for two days either alone (control) or in the presence of RAPA and/or ATRA. The Treg populations were extensively washed prior to mixing with the CD4 T cells to prevent transfer of ATRA or RAPA, or any other soluble suppressive agent. After three days of co-culture, we examined whether the unlabeled activated Tregs could induce FOXP3 expression in the CFSE labeled effector T cells (**Fig 4B**). Only Tregs expanded with RAPA or with RAPA and ATRA were able to induce a suppressor phenotype, as measured by a ∼2-fold increase in the percentage of CFSE-labeled T cells that expressed FOXP3.

Previous reports indicate that the TGF-β propeptide latency-associated peptide (LAP) is involved in mediating infectious tolerance, although alternate mechanisms have been proposed [34], [52]. In order to determine whether LAP expression correlated with the ability of differentially stimulated Tregs to induce infectious tolerance, we analyzed LAP expression by flow cytometry (**Figs 4C and 4D**). We observed that Tregs cultured in the presence of RAPA expressed high levels of LAP relative to Tregs cultured under control conditions, while in Tregs expanded in the presence of ATRA, cell surface expression of LAP dropped dramatically. Tregs co-cultured in both ATRA and RAPA expressed intermediate levels of LAP. However, Tregs from these combined RAPA/ATRA co-cultures effectively induced *FOXP3* expression in Teff cells (**Fig 4A**), suggesting that either other factors expressed by RAPA-treated Tregs may contribute to the induction of infectious tolerance, or the reduced amount of LAP present on the ATRA/RAPA Tregs was sufficient to induce infectious tolerance. Together, our data indicates that RAPA expanded Tregs are better equipped to promote infectious tolerance than ATRA-expanded Tregs.

### ATRA and RAPA affect Treg subsets equally

**Figure 5:**
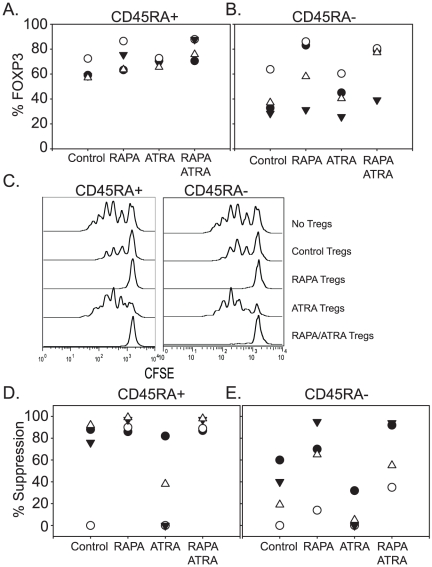
ATRA and RAPA affect Treg subsets equally. CD4+ CD25high CD45RA+ (**A**.) or CD4+ CD25high CD45RA− (**B**.) populations were sort purified and expanded by anti-CD3 Ab-loaded K.64.86 aAPCs in the presence of 100 ng/ml of rapamycin, 10 nM ATRA or a combination of both. After 12–15 days of culture, FOXP3 expression was measured by flow cytometry. Data from 4 unique donors is displayed. **C**. Representative suppression data from a single donor using a 1∶4 Treg to Effector ratio. **D and E**. Compiled suppression data from 4 donors (the donor in C. is the closed triangle).

Since Tregs are a heterogeneous population that can be subdivided by a variety of cell surface markers, including ICOS, CD45RA and CD103 [20], [23], [29], [31], we wanted to know if ATRA was effective in promoting Treg activity in particular subsets but not others, and if the ability of ATRA to promote Treg expansion correlated with the relative abundance of a particular Treg subset. We used CD45RA expression to define Tregs with stable (CD45RA+) and unstable (CD45RA−) phenotypes . Sort-purified populations of CD45RA+ and CD45RA− Tregs were activated and cultured in the presence of RAPA, ATRA, RAPA+ATRA, or no additives (control). We observed that compared to the expanded CD45RA^−^ subset, all expanded CD45RA^+^ populations contained higher percentages of *FOXP3*-positive cells and also demonstrated higher suppressor activity overall (**Fig 5A–E**). However, in one donor, Treg suppressive activity in the CD45RA^+^ population was lost after expansion. Suppressive activity could be restored when these cells were cultured in the presence of RAPA and RAPA+ATRA, but not ATRA alone. We observed a similar trend in the experiments using CD45RA^−^ Tregs. Importantly, when ATRA was used as a single agent, the suppressive activity of natural Tregs was diminished in all cultures tested, suggesting that ATRA could have a detrimental effect on natural Treg function after *ex vivo* expansion. However, it is worth noting that in 3 out of 4 CD45RA^−^ Treg cultures there was more suppressive activity in RAPA+ATRA cultures than in the RAPA alone cultures, suggesting that the addition of ATRA to RAPA may help preserve Treg activity during *ex vivo* expansion. These data highlight the inherent differences of suppressive activity among human Treg subsets. Moreover, they show that as a single agent, ATRA may exert a detrimental effect on expanded Treg function, but in some individuals, ATRA can augment RAPA-mediated Treg suppressive activity.

### Examination of the Treg-specific demethylated region (TSDR) predicts functional activity

**Figure 6:**
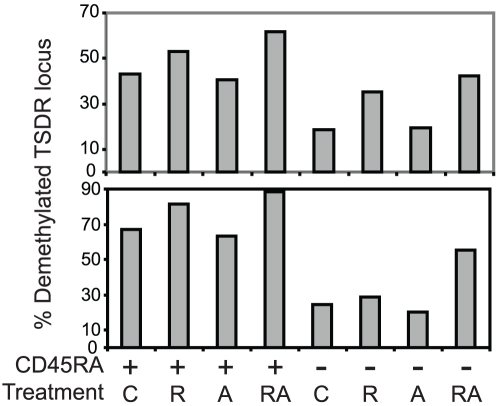
Status of the Treg-specific demethylated region (TSDR) predicts functional activity. Prior to setting up the suppression assays displayed in Fig 5, 1 million expanded T cells were harvested, genomic DNA was prepared, and methylation status of TSDR was determined as described in the Material and Methods. The percentage of demethlyated CpG residues from two donors is displayed. R, A, and RA refers to Tregs expanded in RAPA, ATRA or RAPA and ATRA respectively.

FOXP3 expression alone does not always correlate with human T suppressor activity and as shown in **Fig 2** and our previous work , *in vitro* suppressive assays do not distinguish between transient and sustained Treg suppressive activity. Recently, an association between FOXP3 epigenetic status and suppressive function has been made in both murine and human Tregs [4], [16]. We wished to determine whether epigenetic status of FOXP3 within the previously defined Treg-specific demethylated region (TSDR) correlated with functional activity. Prior to initiating the *in vitro* suppression assay shown in Figure 5, genomic DNA was harvested from Tregs cultured for 15 days in the presence of RAPA, ATRA, ATRA plus RAPA, or no supplements at all. The DNA was subjected to bisulfate conversion and epigenetic analysis to determine the percentage of methylated versus demethylated CpG residues within the TSDR locus (**Fig 6**). We saw a significant correlation between the degree of suppression (Fig 5D–E) and the percentage of demethylated CpG residues (correlation coefficient = 0.535, p = .03), supporting the use of this assay as a rapid means to determine the suppressive potential of expanded Treg populations.

## Discussion

Several animal models have demonstrated the enormous therapeutic potential of Tregs, but only recently have Tregs been used to treat human disease . A key challenge to the field is to develop approaches to generate therapeutic numbers of human Tregs that retain their functionality, trafficking patterns and survival ability once reinfused into the host. We chose to investigate whether the retinoic acid derivative ATRA could promote the expansion of natural Tregs, largely based on its ability to ameliorate autoimmune diseases in mice, including type 1 diabetes [55], experimental allergic encephalomyelitis [36] and colitis [37]. Several groups have investigated how retinoic acid affects the generation of adaptive or inducible (iTregs), especially in the gut environment [14], in order to understand how ATRA alters the progression of autoimmune disease. Interestingly, ATRA appears to promote gut homing of CD4 T cells by inducing CCR9 and α4β7 expression. Gut DCs are the most adept at generating ATRA *in vivo*, causing some to speculate that the ability of ATRA to generate iTregs in the gut is key to maintaining the balance between tolerance and immunity [25]. How ATRA generates iTregs is not completely understood, but the effect appears TGF-β-dependent. By modulating RORγt, it was previously demonstrated that this ATRA target also contributes to FOXP3 upregulation . While TGF-β is required to observe ATRA-mediated effects on FOXP3 expression, it is not clear if in the absence of TGF-β, ATRA enhances Treg functional activity. Our data suggest that ATRA alone may have a deleterious effect on Treg functional activity, but in combination with RAPA, ATRA may promote the expansion of Tregs with durable activity in the absence of exogenously added TGF-β.

Because of the difficulty in isolating a pure population of Tregs and ability of ATRA to augment FOXP3 expression, some groups have investigated whether effector T cells could be converted to Tregs using ATRA and TGF-β as a means of generating large numbers of therapeutic Tregs . The obvious advantage of this approach is that it would greatly reduce the number of GMP reagents required to purify the starting cell population and would greatly simplify the Treg expansion process. Most studies performed in mice report robust *in vivo* suppressive activity of TGF-β-induced Tregs in the treatment of colitis [22] and EAE [41]. This suppressive activity should be further enhanced by culture in the presence of ATRA . However, it is not clear from these studies how long these induced Tregs needed to maintain their suppressive phenotype to alter the disease course. A possible flaw in the TGF-β/ATRA-based expansion strategy outlined above is that T cells have a remarkable ability to modulate their function in response to the surrounding cytokine and costimulatory milieu. While this post thymic iTreg development helps the immune system to tailor its effector mechanism to target bacteria, viruses or worms, it can be subverted to serve as an immune escape mechanism if a particular tumor or infectious agent can skew an antigen-specific T cell response from an effector to a regulatory phenotype. Thus, ATRA/TGF-β-cultured cells may appear to be Tregs following *ex vivo* expansion, but it is unclear whether they will retain suppressive activity following re-infusion. For instance, in a GVHD model, iTregs failed to retain FOXP3 expression and suppressive activity following reinfusion into the host [26]. These data are reminiscent of the finding that purified central memory T cells, *ex vivo* expanded under conditions designed to endow them with an effector memory phenotype at the end of the culture period, reverted and functioned as central memory cells when infused into their hosts [9]. This suggests that T cells have a mechanism to “remember” their phenotype prior to *ex vivo* expansion, cautioning against the use of iTregs for indications requiring long-term immunosuppression.

A limited number of studies have asked whether these same principles of iTreg generation are conserved in human T cells. While one study noted that ATRA could induce FOXP3 in the absence of exogenous TGF-β, it wasn't clear whether the media used to expand these Tregs was TGF-β-free . A subsequent study showed that indeed TGF-β was required for FOXP3 induction, but this process was limited to naïve T cells . In this report, human iTregs generated by culture of CD4^+^CD25^−^ T cells in the presence of ATRA and TGF-β had *in vitro* suppressive activity and other phenotypic characteristics of Tregs. This group also examined the effect of ATRA/TGF-β treatment on the expansion of enriched natural Tregs. They found that ATRA/TGF-β did not hinder natural Treg growth, resulted in higher, more persistent levels of FOXP3 expression, and generated Tregs with superior suppressive activity at the end of two weeks of culture. As a result, this group advocated the use of ATRA to expand natural as well as adaptive Tregs. We were able to largely replicate these findings using natural Tregs but came to the opposite conclusion regarding the utility of expanding natural Tregs in the presence of ATRA. Prolonged *ex vivo* expansion revealed to us that the effects of ATRA on natural Tregs was transient, and did not result in the generation of a durable Treg population. Moreover, ATRA treatment did not suppress cytokine production in co-cultured FOXP3− T cells (Fig 4), nor did it lead to upregulation of membrane bound TGF-β (Fig 4), suggesting that Tregs expanded in this manner were not well equipped to promote infectious tolerance. Lastly, ATRA treatment did not enrich for T cells with a demethylated TSDR, which others have shown to be strongly correlated with long-term maintenance of the Treg suppressive phenotype [28].

Our studies examining the effect of ATRA on human Tregs are reminiscent of studies of the effect of TGF-β on Tregs , and in fact these findings may be interrelated. In mice, TGF-β appears to induce FOXP3 expression and a suppressive phenotype in effector CD4 T cells. In contrast, when human CD4 T cells are treated with TGF-β, FOXP3 is induced but limited suppressive activity is detected . Our studies indicate that ATRA is able to potentiate the ability of TGF-β treated human CD4 T cells to impersonate human T regulatory cells but ATRA as a single agent is unable to promote the generation of Tregs that are attractive for use in therapeutic applications. The interplay between TGF-β, ATRA, DNA methylation and T cell suppression is unclear. In some models, ATRA induces the activation of latent TGF-β [11], but it is unknown whether a similar mechanisms occurs in T cells. Moreover, ATRA can bind a whole family of retinoic acid receptors that can lead to a wide array of outcomes, some of which promote immunity while others promote tolerance [54]. Thus, understanding how ATRA hinders expansion of natural Tregs whereas the combination of ATRA and RAPA enhances the expansion of natural Tregs remains an open question.

While our studies indicate that ATRA is unsuitable as a single agent for expansion of human Tregs, a case can be made for the combined use of RAPA and ATRA. ATRA did not interfere with RAPA's ability to promote infectious tolerance (Fig 4A–C) and only modestly diminished LAP expression (Fig 4D). Importantly, 3 out of 4 CD45RA− Treg cultures grown in the presence of ATRA and RAPA had higher suppressive activity than those expanded in RAPA alone, suggesting that in the absence of TGF-β, ATRA may potentiate RAPA's effect on Treg stability and function. Moreover, the use of ATRA during the *ex vivo* expansion of Tregs destined to treat autoimmune diseases of the gut may be particularly attractive, since ATRA upregulates expression of CCR9 and other factors that promote migration into the gut . Importantly, the use of RAPA and ATRA in Treg culture increased the percentage of Tregs with demethylated FOXP3 alleles (Fig 6) suggesting that these Tregs are more likely to remain as Tregs once re-infused in the patient and provide long lasting and effective control of autoimmune disease.

## Materials and Methods

### Cell Isolation and Purification

Mononuclear cell enriched apheresis products were obtained by leukapheresis of healthy volunteer donors by the University of Pennsylvania Human Immunology Core. All specimens were collected under a University Institutional Review Board-approved protocol, and informed written consent was obtained from each donor. CD4^+^ T cells were purified from the apheresis product using RosetteSep human CD4+ T cell enrichment cocktail (Stemcell Technologies, Vancouver, Canada). CD127dim, CD25high T regulatory cells were sorted using a MoFlo high-speed cell sorter (DakoCytomation). Where indicated, CD127dim, CD25high, CD45RA^+^ and CD127dim, CD25high, CD45RA− populations of Tregs were also sorted.

### Stimulation and Expansion of Human T Cells

K.64.86, the K562-based artificial antigen presenting cell line expressing CD64 (to enable loading of anti-CD3 Abs) and CD86, was previously described [42]. K64.86 aAPCs were washed and resuspended in serum-free culture medium (X-VIVO 15 medium, LONZA, Walkersville, MD) 24 hours prior to antibody loading. After 100Gy of irradiation, washing, and addition of 1 µg/ml anti-CD3 Ab, the aAPCs were rotated at 4°C for 30 min, after which unbound antibody was removed by washing three times. The Ab-loaded K64.86 cells were resuspended in serum-free culture medium at a density 1×10^6^ cells/ml, and combined with human T cells (also in serum-free medium) at a final ratio of 1 K64.86 cell: 2 CD4 cells. Where indicated, rapamycin (Calbiochem, San Diego, CA) was added on day 0 to a final concentration of 100 ng/ml. All-trans-retinoic acid (Sigma-Aldrich, St. Louis, MO) (10 nM for serum-free conditions and 10 µM for serum-containing conditions) was added on day 0. Where indicated, human AB serum (Valley Biomedical, Winchester, Virginia) was added to a final concentration of 10% after 24 hrs of culture, while on day 2 of culture, human IL-2 (Chiron Therapeutics, Emeryville, CA) was added to a final concentration of 300 IU/ml. Cultures were monitored for cell volume and cell density using a Coulter Multisizer 3 (Beckman Coulter, Fullerton, CA) on days 5, 8, 12 and 15 of culture. Following counting, the cell concentration was adjusted to 3×10^5^ T cells/ml by adding fresh medium supplemented with 300U/ml IL-2 in the presence of ATRA and/or rapamycin, where indicated.

### *In Vitro* Suppression Assay

The suppression assay was performed as previously described . Briefly, various ratios of expanded Tregs and CFSE− labeled autologous PBMCs were mixed with anti-CD3 Ab-coated beads (Invitrogen Dynal AS, Oslo, Norway) using 1 bead per cell in 100 µl of media in round bottom 96 well plates (Corning Incorporated, Corning, NY). Cultures were harvested 4 days later and stained with APC-conjugated anti-CD8 antibodies (BD Pharmingen, San Diego, CA). Data were acquired on a FACSCalibur (BD Biosciences) flow cytometer using Cell Quest Pro software and analyzed using FlowJo software (Tree Star Inc., Ashland, OR). For quantitative analysis of Treg suppression capacity, the gates were placed on CD8+ CFSE+ cells. The number of cells in each generation was determined using FlowJo software. The percentage of undivided (PU) cells was calculated as the percentage of cells in generation 0 at the end of the assay: PU = N (0)/(N (0)+N (1)+…+N (m))×100. Fold expansion (FE) was calculated as the number of offspring cells at the end of the assay divided by the number of cells at the beginning of the assay: FE = (N (1)+N (2)+…+N (m))/N estimated (0), where N estimated (0) = N (0)+N (1)/2+N (2)/4+…+N (m−1)/2^m−1^.

### Antibodies, Surface and Intracellular Staining

Anti-CD4-APC (555349), anti-CD8-APC (555369), anti-CD25-PE (555432), anti-CD27-PE (555441), anti-CD62L-APC (559772), anti-CD64-FITC (555527), anti-CD127-PE (557938), were obtained from BD Biosciences; anti-FOXP3-AlexaFluor 488 and anti-FOXP3-Pacific Blue antibodies were purchased from Biolegend (San Diego, CA), anti-LAP-PE antibodies (FAB2463P) were purchased from R&D Systems (Minneapolis, MN). Intracellular FOXP3 staining was performed using the FOXP3 Fix/Perm Buffer set (Biolegend) according to the manufacturer's recommendations. The intracellular cytokine assay was performed as previously described [47].

### DNA isolation and methylation analysis

Following *in vitro* T cell expansion, genomic DNA from 500,000 T cells was isolated using the DNeasy Kit (Qiagen, Germantown, MD) per the manufacturer's recommendations. Quantification of the degree of methylation at the *FOXP3*-TSDR locus was conducted by real-time PCR method and performed by Epiontis (Berlin, Germany) as previously described [49].

### Statistical Analysis

Statistical analysis was performed using the indicate test via SigmaPlot v.11 software. P values equal to or less than 0.05 were considered significant.
